# The Potential Role of Reactive Oxygen Species Produced by Low-Density Neutrophils in Periodontitis

**DOI:** 10.1055/s-0044-1782211

**Published:** 2024-05-14

**Authors:** Ali Omran Mousa, Ali Hussien Abass Al Hussaini, Hashim Mueen Hussein

**Affiliations:** 1 Department of Periodontics, College of Dentistry, University of Baghdad, Baghdad, Iraq; 2 Department of Oral and Maxillofacial Surgery, College of Dentistry, University of Baghdad, Baghdad, Iraq; 3 Department of Conservative Dentistry, College of Dentistry, Mustansiriyah University, Baghdad, Iraq

**Keywords:** periodontitis, reactive oxygen species, low-density neutrophils

## Abstract

**Objective** Neutrophils own an arsenal of dischargeable chemicals that enable them to handle bacterial challenges, manipulating innate immune response and actual participation in acquired immunity. The reactive oxygen species (ROS) are one of the most important chemicals that neutrophils discharge to eradicate pathogens. Despite their beneficial role, the ROS were strongly correlated to periodontal tissue destruction. Lowdensity neutrophils (LDN) have been recognized for producing enhanced quantities of ROS. However, the potential role of ROS produced by LDN in periodontitis is unknown. The aim of the study was to investigate the impact of ROS produced by LDN in periodontal diseases.

**Materials and Methods** Venous blood and periodontal parameters were obtained from 100 systemically healthy subjects divided into 40 participants with healthy periodontium in the control group and 60 with unstable periodontitis in the study group. Flow cytometry was used to measure the production of ROS by LDN in both groups.

**Statistical Analysis** The data were analyzed for normal distribution using the Shapiro-Wilk test at *p* < 0.05, Spearman's correlations, and Mann-Whitney U test. Statistical analysis was performed in SPSS v25.

**Results** No difference between the groups had been obtained in ROS production by LDN. However, a significant positive correlation existed between ROS and clinical attachment loss in periodontitis.

**Conclusion** LDN exhibits the same ROS generation capacity in the control and periodontitis groups.

## Introduction

Periodontitis is a multifactorial progressive condition characterized by continuous destruction of periodontal tissue under the burden of a dysbiotic microflora, host immune response, environmental factors, and subject genetic susceptibly. 1 2 Periodontitis encompasses the existence of inflammation in the periodontal tissues, the development of periodontal pockets, a breakdown of connective tissue, alveolar bone loss, and potential tooth loss. 3 4 5 6 7 Despite their beneficial role in eradicating pathogens, inflammation is a double-edged sword; it is considered a crucial source of periodontal tissue destruction, involving leukocytes, complement, and reactive oxygen species (ROS). 8 ROS seem to be short-lived, extremely reactive oxygen-reduced radicals, including superoxide  , hydrogen peroxide (H _2_ O _2_ ), the hydroxyl radical (OH), and singlet oxygen. 9 At the cellular level, ROS is crucial for eukaryotic cell physiologic functions such as signaling transmission, differentiation, apoptotic cell death, 8 9 and pathogen oxidative death. 10

A clinical study revealed that serum reactive oxygen metabolites were positively correlated with immunoglobulin G antibodies to keystone periodontal pathogens such as *Porphyromonas gingivalis* , *Aggregatibacter actinomycetemcomitans* , and *Prevotella intermedia* . 11 12

Furthermore, the level of ROS markedly regulates many biological events of most cellular components in the periodontium. For instance, a reduced ROS level decreases bone loss by downregulating the osteoclast differentiation marker genes; conversely, it enhances the proliferation and differentiation of human periodontal ligament fibroblasts (hPDLFs) and osteoblasts. 13

Low-density neutrophils (LDN) are a unique attention-seeking neutrophil phenotype strongly associated with various immunological diseases, endocrine disorders, systemic diseases, and infections. 10 Based on previous studies on healthy subjects, the LDN exhibits enhanced ROS-generating capacity compared to the normal-density neutrophil. 10

To the best of our knowledge, the potential impact of ROS produced by LDN in the periodontitis has not been studied yet. However, this study tries to illustrate this role in the pathogenesis of periodontitis using a multicolor flow cytometry.

## Materials and Methods

### Study Design

This observational research was conducted from August 2022 to March 2023 at the College of Dentistry, University of Baghdad.

### Ethical Approval

All procedures used in this study adhered to the guidelines outlined in the Helsinki Declaration of 1964 and its subsequent revisions, specifically concerning research involving human subjects. The protocol for this study has been authorized by the Ethics Committee of the College of Dentistry at the University of Baghdad. The reference number for this approval is 450, the project number is 450622, and the approval date is January 19, 2021. Following the provision of comprehensive information elucidating the nature and objectives of the research, every participant was requested to affix their signature on an informed consent document. Following the participants' agreement, a clinical examination was conducted, and then blood samples were collected from each individual.

### Inclusion Criteria

One hundred systemically healthy participants were allocated into two groups:

- The control group consisted of 40 subjects with healthy periodontium defined as bleeding on probing (BOP) less than 10% and periodontal pocket depth (PPD) ≤3 mm. Additionally, there was no evidence of attachment loss during periodontal probing. 14
- The periodontitis group consisted of 60 subjects with unstable periodontitis, defined as BOP greater than 10%, with clinical attachment loss (CAL) at the interproximal area at ≥2 nonadjacent teeth or two teeth with buccal or oral CAL of 3 mm with pocketing greater than 3 mm. 15

### Exclusion Criteria

Patients with dental implants, systemic or oral autoimmune illness, infections, inflammatory diseases, drug intake (antibiotics, nonsteroidal anti-inflammatory drugs) within 3 months, endocrine disorders, systemic problems, pregnancy, and smoking habits were excluded. The wisdom teeth were excluded from periodontal examinations.

### Subjects Eligibility

**Fig. 1:**
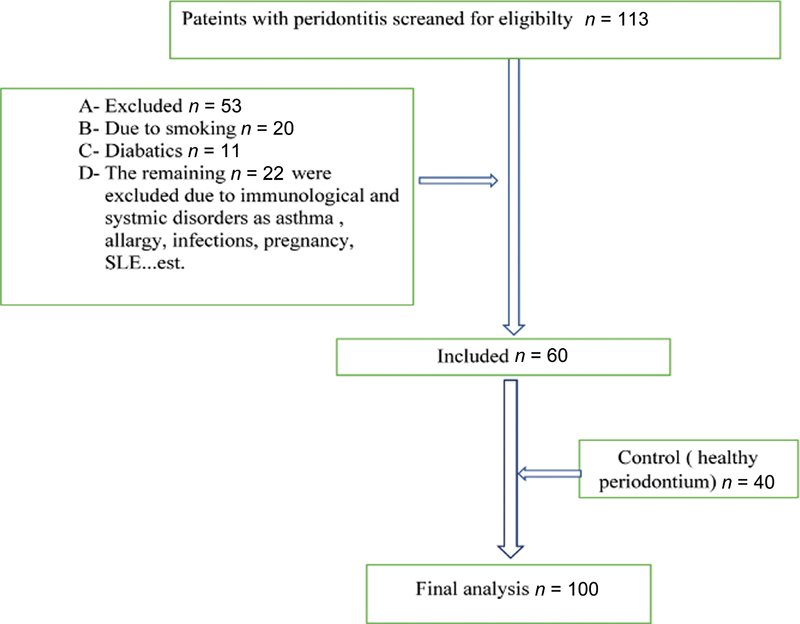
Subject eligibility chart flow.

Patients with periodontitis referred for periodontal therapy were initially screened ( *n* = 113) to evaluate their eligibility for recruitment. After applying the inclusion/exclusion criteria, 53 patients were excluded for different reasons, and 60 patients were included in the final analysis. Later, the patients with healthy periodontium were included ( *n* = 40) as controls ( Fig. 1 ).

### Clinical Examination

All clinical examinations were done utilizing the Michigan O probe (Osung USA, Houston, United States) with markings at 1, 2, 3, 5, 7, 8, 9, and 10 mm. Six surfaces were examined to estimate BOP%, PPD, and CAL: mesiobuccal, middle buccal, distobuccal, mesio-oral, middle oral, and disto-oral. In contrast, plaque index (PI) 16 was estimated by examination of four surfaces (mesial, distal, buccal, and oral).

### Blood Collection

After clinical examination and subject allocation, 5 mL of intravenous blood was collected and placed in a 10-mm ethylenediaminetetraacetic acid (EDTA) tube. The collected blood was preserved at 4°C protected from light for 30 minutes. LDN purification must done within a period not exceeding 6 hours to purify pure, healthy, and activated LDN. 10

### Purification of LDN

**Fig. 2:**
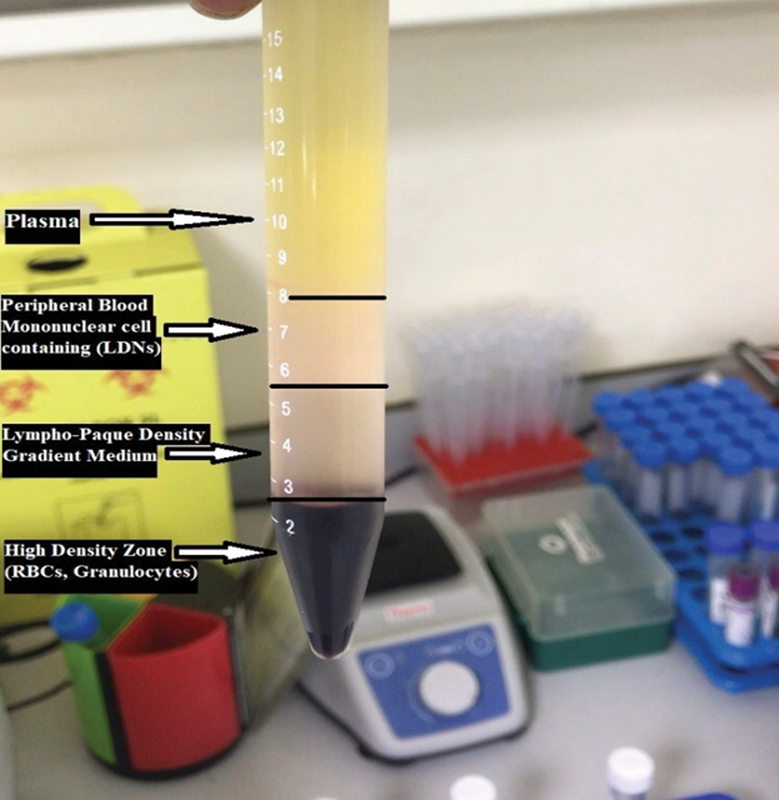
Purification of low-density neutrophils (LDN) in peripheral blood mononuclear cells (PBMCs).

The LDN were purified by mixing 5 mL of anticoagulated blood with 2 mL of 6% dextran T500 by inversion and allowing it to rest for 45 minutes to precipitate red blood cells. Next, the layer of leukocyte-rich plasma was placed carefully on top of 5-mL Lympho-Paque at 1.077 g/mL density. The sample was centrifuged at 520 *g* for 20 minutes at 4°C using a Thermo Scientific Megafuge 8R Small Benchtop Centrifuge. A layer of mononuclear cells (MNC), including monocytes, lymphocytes, and LDNs, is located at the plasma-lympho-paque interface ( Fig. 2 ). LDN-containing MNC was collected, diluted 1:2 with phosphate buffer saline (PBS), and stored in ice until use.

### Measurement of Reactive Oxygen Species

**Fig. 3:**
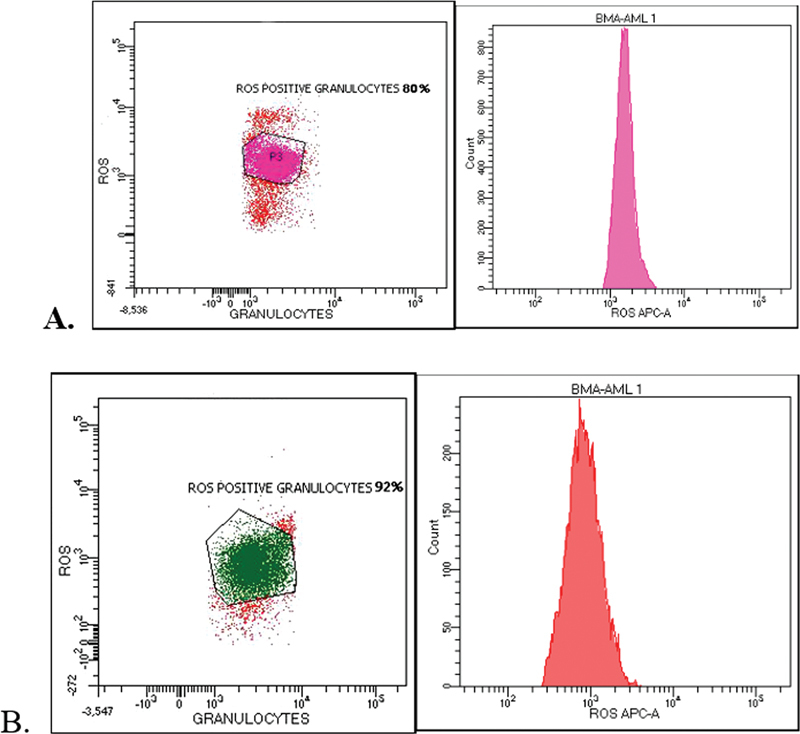
Reactive oxygen species (ROS) expression via LDN in ( **A** ) a healthy periodontium. ( **B** ) Periodontitis.

ROS production was measured by detecting fluoresce changes in LDNs loaded with dihydrorhodamine 123 (DHR-123). Cells (1 × 10 ^6^ ) were suspended in 100 mL of 15 mM DHR-123 in PBS and incubated for 15 minutes at 37°C in the dark. Cells were washed with 1 mL PBS, resuspended in 100 mL of PBS containing 20 nM of Probol-12-myristate-13-acetate (PMA), and incubated at 37°C in the dark for 50 minutes. Next, cells were washed in cold PBS and resuspended in 1% paraformaldehyde in PBS. Finally, cells were kept in a cold and dark place until analyzed by flow cytometry. The cells were gated by a dot-plot analysis, and 10,000 cells were acquired per sample. The cells were analyzed in BD FACSCanto II multicolor flow cytometry with the BD FACSDiva Software v9.0 ( Fig. 3 ).

## Results

### Descriptive Data

In the control group, the descriptive data show the minimum, maximum, mean, and standard deviation of different variables.

The descriptive data in the periodontitis group showed the minimum, maximum, mean, and standard deviation of different variables (as shown in Table 1 ).

**Intergroup comparisons** showed a significantly higher BOP and PI in the periodontitis group than in the control group ( *p* < 0.01) (as shown in Table 2 ).

However, no significant difference was observed in ROS production between the two groups (as shown in Table 3 ).

### Correlations

#### Correlations in the Control Group

The observed data shows a significant positive correlation between BOP% and PI in the control group. However, no correlation was observed between ROS and the other variables in the control group ( Table 4 ).

#### Correlations in the Periodontitis Group

The collected data show a significant positive correlation among all periodontal parameters; however, ROS was significantly and positively correlated with CAL in the periodontitis group ( *p* = 0.275; 2-tailed = 0.034) ( Table 5 ).

## Discussion

Neutrophils are the predominant leukocytes in circulation and are considered the main cellular arm of innate immunity, which responds to inflammation and infection. 17 In inflamed tissues, neutrophils perform several antimicrobial tasks, 18 such as degranulation, 19 ROS generation, 20 phagocytosis, and neutrophil extracellular trap (NET) formation. 21 In addition to innate immunological activities, neutrophils modulate adaptive immune responses. 22 In fact, neutrophils do not appear to be a homogeneous population that often behaves similarly; however, multiple subpopulations of neutrophils have been proposed in diverse health and disease situations. 23

LDN is a unique neutrophil phenotype that exists in large numbers in various pathological disorders like inflammations, infections, cancer, and immunosuppression. 24 Based on a previous study, LDN has also been detected in the peripheral blood of systemically healthy subjects and exhibits an enhanced capacity for ROS compared to the normal high-density neutrophil (HDN). 10

Interestingly, the outcomes of our study revealed no significant difference in ROS produced via LDN between the study groups, which may indicate that LDN is an excellent ROS producer regardless of the periodontal status. Despite this, LDN exhibits more ROS production capacity in the periodontitis group at 89% compared to the healthy control group at 85%. However, this may be due to the systemic dissemination of periodontal pathogens and their products via the ulcerated pocket epithelium. 25 26

Additionally, a significant positive correlation was observed between ROS and CAL in the periodontitis group. Based on this outcome in healthy subjects, ROS is beneficial in pathogen eradication and differentiation of cells. 11 On the other hand, in periodontitis patients, increasing ROS coincided with increasing destruction of the periodontal tissue (CAL). In conclusion, enhanced ROS benefits the healthy periodontium; in contrast, it is harmful in periodontitis.

However, the schizophrenic behavior of ROS in the current study could be related to the homeostatic imbalance between ROS and antioxidant defense systems, 27 28 as the periodontitis patients showed lower plasma and serum total antioxidant concentrations (TAOC) levels than healthy controls. 28 29

The TAOC is the main body defense system for neutralizing ROS. Furthermore, decreased TAOC and elevated ROS might be risk factors for periodontitis or might be induced by periodontal inflammation. 30 However, to estimate the precise effect of TAOC, the analysis of site-specific LDNs purified from saliva, gingival crevicular fluid (GCF), and the junctional epithelium is mandatory. Indeed, in the murine model, the LDN can be characterized by the expression of CD11b and Ly6G +  markers. 31 However, in humans, the absence of Ly6G+ antigen that distinguishes normal-density neutrophils (NDN) from LDN makes site-specific studies with high overlap effects between these neutrophil subpopulations. 32 33 34 Consequently, the density gradient centrifugation of peripheral blood is the sole method for the purification of LDN. The above-mentioned considerations present a major challenge to accurately evaluate the actual impact of TAOC in neutralizing LDN ROS.

## Conclusion

Irrespective of the periodontal status, LDN demonstrates an increased capability for generating ROS. The enhanced ROS benefits the healthy periodontium; conversely, it is harmful in periodontitis.

**Table 1:** Descriptive data in the control group

	Minimum	Maximum	Mean	Std. deviation
Age (Year)	21	55	34.5	±9.94
BOP %	0.021	0.091	0.0682	±.0227
PI (%)	0.111	0.511	0.37171	±.115251
ROS	0.44	0.99	0.8498	±.14648

Abbreviations: BOP, bleeding on probing; PI, plaque index; ROS, reactive oxygen species.

**Table 2:** Descriptive data in the periodontitis group

	Minimum	Maximum	Mean	Standard deviation
Age (Year)	21	57	36.28	±9.372
BOP %	0.111	0.881	0.405	±.2247
PI (%)	0.31	2.5	1.28	±.57
PPD (mm)	4.1	9.1	5.51	±1.279
CAL (mm)	1.1	11.1	5.26	±2.6670
ROS	0.410	0.99	0.89268	±.111128

Abbreviations: BOP, bleeding on probing; CAL, clinical attachment loss; PI, plaque index; PPD, periodontal pocket depth; ROS, reactive oxygen species.

**Table 3:** The intergroup comparison

Biomarker	Mean control	Mean periodontitis	*p* -value*
Age (Year)	34.48	36.28	0.430 (NS)
BOP %	0.0682	0.405	0.000*
PI (%)	0.37171	1.28	0.000*
ROS	0.8498	0.89268	0.320 (NS)

Abbreviations: BOP, bleeding on probing; NS: non significant at *P* -value ≥0.05; PI, plaque index; ROS, reactive oxygen species.

* significant at *P* -value <0.05.

**Table 4:** The correlations in the control group

	Spearman correlation	Age	BOP %	PI	ROS
Age	Correlation coefficient	1.000			
	Sig. (2-tailed)				
BOP %	Correlation coefficient	0.052	1.000		
	Sig. (2-tailed)	0.748			
PI	Correlation coefficient	–0.131	0.689 a	1.000	
	Sig. (2-tailed)	0.419	0.000 *		
ROS	Correlation coefficient	0.004	0.094	0.238	
	Sig. (2-tailed)	0.979	0.565	0.139	

Abbreviations: BOP, bleeding on probing; NS: non significant at *P* -value ≥0.05; PI, plaque index; ROS, reactive oxygen species.

a Refers to the significant correlation coefficient.

* significant at *P* -value <0.05.

**Table 5:** The correlations in the periodontitis group

	Spearman correlation	Age (Year)	BOP %	PI	PPD	CAL	ROS
Age (Year)	Correlation coefficient	1.000					
	Sig. (2-tailed)						
BOP %	Correlation coefficient	0.141	1.000				
	Sig. (2-tailed)	0.284					
PI (%)	Correlation coefficient	0.175	0.808 a	1.000			
	Sig. (2-tailed)	0.180	0.000				
PPD (mm)	Correlation coefficient	0.303 b	0.808 a	0.653 a	1.000		
	Sig. (2-tailed)	0.019	0.000	0.000			
CAL (mm)	Correlation coefficient	0.176	0.492 a	0.442 a	0.532 a	1.000	
	Sig. (2-tailed)	0.179	0.000	0.000	0.000		
ROS	Correlation coefficient	0.280 b	–0.015	0.094	0.160	0.275 b	
	Sig. (2-tailed)	0.030	0.908	0.473	0.222	0.034	

Abbreviations: BOP, bleeding on probing; CAL, clinical attachment loss; PI, plaque index; PPD, periodontal pocket depth; ROS, reactive oxygen species.

a Refers to the significant correlation coefficient.

b Refers to the Sig. (2-tailed).

## References

[1] Saliem S S Bede S Y Cooper P R Abdulkareem A A Milward M R Abdullah B H Pathogenesis of periodontitis: a potential role for epithelial-mesenchymal transition Jpn Dent Sci Rev 2022 58 268 278 36159185 10.1016/j.jdsr.2022.09.001 PMC9489739

[2] Mahmood A A Abbas R F Assessment of NLRP3 gene polymorphisms with periodontitis as compared with healthy periodontium in Iraqi Arabs patients Eur J Dent 2023 17 04 1338 1348 36812929 10.1055/s-0043-1761185 PMC10756796

[3] Hussein H M Mahmood A A Alberaqdar F A The prevalence and relationship of root caries depth and gingival recession among different Iraqi groups Mustansiria Dent J 2015 12 01 144 155

[4] Saeed N A Hussein H M Mahmood A A Prevalence of dental anxiety in relation to sociodemographic factors using two psychometric scales in Baghdad Mustansiria Dent J 2017 14 01 38 50

[5] Mahmood H K Al-Ghurabi B H Low frequency of active HCMV infection among chronic periodontitis patients Biochem Cell Arch 2020 20 01 847 851

[6] Mulawarmanti D Parisihni K The effect of *Sticopus hermanii* -hyperbaric oxygen therapy to inflammatory response of diabetic periodontitis Bristol, UK IOP Publishing 2019

[7] Mousa A O Saliem S S Abdullah B H Raad Abdulbaqi H Age gender and site effect on immunohistochemical expression of TGF-β1 and IFN-γ in hereditary gingival fibromatosis J Glob Pharma Technol 2019 11 02 542 547

[8] McClean C Harris R A Brown M Brown J C Davison G W Effects of exercise intensity on postexercise endothelial function and oxidative stress Oxid Med Cell Longev 2015 2015 723679 26583061 10.1155/2015/723679 PMC4637109

[9] Di Meo S Reed T T Venditti P Victor V M Role of ROS and RNS sources in physiological and pathological conditions Oxid Med Cell Longev 2016 2016 1.245049E6 10.1155/2016/1245049 PMC4960346 27478531

[10] Blanco-Camarillo C Alemán O R Rosales C Low-density neutrophils in healthy individuals display a mature primed phenotype Front Immunol 2021 12 672520 34276661 10.3389/fimmu.2021.672520 PMC8285102

[11] Budhy T I Arundina I Surboyo M DC Halimah A N The effects of rice husk liquid smoke in *Porphyromonas gingivalis* -induced periodontitis Eur J Dent 2021 15 04 653 659 34041725 10.1055/s-0041-1727554 PMC8630964

[12] Abdulhameed V S Saliem S S Hassan T A Evaluation of crestal bone loss and alkaline phosphatase level in saliva according to different flap designs in single-tooth dental implant surgery (a clinical comparative study) Biomed Pharmacol J 2017 10 04 1863 1869

[13] Baser U Gamsiz-Isik H Cifcibasi E Ademoglu E Yalcin F Plasma and salivary total antioxidant capacity in healthy controls compared with aggressive and chronic periodontitis patients Saudi Med J 2015 36 07 856 861 26108592 10.15537/smj.2015.7.11954 PMC4503907

[14] Chapple I LC Mealey B L Van Dyke T E Periodontal health and gingival diseases and conditions on an intact and a reduced periodontium: consensus report of workgroup 1 of the 2017 World Workshop on the Classification of Periodontal and Peri-Implant Diseases and Conditions J Periodontol 2018 89 01 S74 S84 29926944 10.1002/JPER.17-0719

[15] Tonetti M S Greenwell H Kornman K S Staging and grading of periodontitis: Framework and proposal of a new classification and case definition J Periodontol 2018 89 01 S159 S172 29926952 10.1002/JPER.18-0006

[16] Silness J Löe H Periodontal disease in pregnancy. II. Correlation between oral hygiene and periodontal condition Acta Odontol Scand 1964 22 01 121 135 14158464 10.3109/00016356408993968

[17] Fine N Tasevski N McCulloch C A Tenenbaum H C Glogauer M The neutrophil: constant defender and first responder Front Immunol 2020 11 571085 33072112 10.3389/fimmu.2020.571085 PMC7541934

[18] Nauseef W M Neutrophils, from cradle to grave and beyond Immunol Rev 2016 273 01 5 10 27558324 10.1111/imr.12463 PMC5001163

[19] Lacy P Eitzen G Control of granule exocytosis in neutrophils Front Biosci 2008 13 01 5559 5570 18508605 10.2741/3099

[20] Zeng M Y Miralda I Armstrong C L Uriarte S M Bagaitkar J The roles of NADPH oxidase in modulating neutrophil effector responses Mol Oral Microbiol 2019 34 02 27 38 30632295 10.1111/omi.12252 PMC6935359

[21] Papayannopoulos V Neutrophil extracellular traps in immunity and disease Nat Rev Immunol 2018 18 02 134 147 28990587 10.1038/nri.2017.105

[22] Leliefeld P H Wessels C M Leenen L P Koenderman L Pillay J The role of neutrophils in immune dysfunction during severe inflammation Crit Care 2016 20 73 27005275 10.1186/s13054-016-1250-4 PMC4804478

[23] Nauseef W M Borregaard N Neutrophils at work Nat Immunol 2014 15 07 602 611 24940954 10.1038/ni.2921

[24] Scapini P Marini O Tecchio C Cassatella M A Human neutrophils in the saga of cellular heterogeneity: insights and open questions Immunol Rev 2016 273 01 48 60 27558327 10.1111/imr.12448

[25] Ohki T Itabashi Y Kohno T Detection of periodontal bacteria in thrombi of patients with acute myocardial infarction by polymerase chain reaction Am Heart J 2012 163 02 164 167 22305832 10.1016/j.ahj.2011.10.012

[26] Abdulkareem A A Al-Taweel F B Al-Sharqi A JB Gul S S Sha A Chapple I LC Current concepts in the pathogenesis of periodontitis: from symbiosis to dysbiosis J Oral Microbiol 2023 15 01 2.197779E6 10.1080/20002297.2023.2197779 PMC10071981 37025387

[27] Kanzaki H Shinohara F Kajiya M Kodama T The Keap1/Nrf2 protein axis plays a role in osteoclast differentiation by regulating intracellular reactive oxygen species signaling J Biol Chem 2013 288 32 23009 23020 23801334 10.1074/jbc.M113.478545 PMC3743476

[28] Chapple I LC Brock G Eftimiadi C Matthews J B Glutathione in gingival crevicular fluid and its relation to local antioxidant capacity in periodontal health and disease Mol Pathol 2002 55 06 367 373 12456773 10.1136/mp.55.6.367 PMC1187272

[29] Patil V S Patil V P Gokhale N Acharya A Kangokar P Chronic periodontitis in type 2 diabetes mellitus: oxidative stress as a common factor in periodontal tissue injury J Clin Diagn Res 2016 10 04 BC12 BC16 10.7860/JCDR/2016/17350.7542 PMC4866088 27190790

[30] Liu C Mo L Niu Y Li X Zhou X Xu X The role of reactive oxygen species and autophagy in periodontitis and their potential linkage Front Physiol 2017 8 439 28690552 10.3389/fphys.2017.00439 PMC5481360

[31] Daley J M Thomay A A Connolly M D Reichner J S Albina J E Use of Ly6G-specific monoclonal antibody to deplete neutrophils in mice J Leukoc Biol 2008 83 01 64 70 17884993 10.1189/jlb.0407247

[32] Damuzzo V Pinton L Desantis G Complexity and challenges in defining myeloid-derived suppressor cells Cytometry B Clin Cytom 2015 88 02 77 91 25504825 10.1002/cyto.b.21206 PMC4405078

[33] Rosales C Neutrophil: a cell with many roles in inflammation or several cell types? Front Physiol 2018 9 113 29515456 10.3389/fphys.2018.00113 PMC5826082

[34] Dumitru C A Moses K Trellakis S Lang S Brandau S Neutrophils and granulocytic myeloid-derived suppressor cells: immunophenotyping, cell biology and clinical relevance in human oncology Cancer Immunol Immunother 2012 61 08 1155 1167 22692756 10.1007/s00262-012-1294-5 PMC11028504

